# Overexpression and biophysical and functional characterization of a recombinant FGF21

**DOI:** 10.1016/j.bpr.2025.100198

**Published:** 2025-01-29

**Authors:** Phuc Phan, Jason Hoang, Thallapuranam Krishnaswamy Suresh Kumar

**Affiliations:** 1 Department of Chemistry and Biochemistry, Fulbright College of Art and Sciences, University of Arkansas, Fayetteville, Arkansas

## Abstract

Fibroblast growth factor 21 (FGF21) is an endocrine FGF that plays a vital role in regulating essential metabolic pathways. FGF21 increases glucose uptake by cells, promotes fatty acid oxidation, reduces blood glucose levels, and alleviates metabolic diseases. However, detailed studies on its stability and biophysical characteristics have not been reported. Herein, we present the overexpression, biophysical characterization, and metabolic activity of a soluble recombinant FGF21 (rFGF21). The far-UV circular dichroism spectra of rFGF21 show a negative trough at 215 nm, indicating that the protein’s backbone predominantly adopts a β sheet conformation. rFGF21 shows intrinsic tyrosine fluorescence at 305 nm. Thermal denaturation using differential scanning calorimetry reveals that rFGF21 is relatively thermally unstable, with a melting temperature of 46.8°C (±0.1°C). The urea-induced unfolding of rFGF21 is rapid, with a chemical transition midpoint of 0.4 M. rFGF21 is readily cleaved by trypsin in limited trypsin digestion assays. Isothermal titration calorimetry experiments show that rFGF21 does not bind to heparin. Interestingly, rFGF21 demonstrates proliferative activity in NIH/3T3 fibroblasts and enhances mitochondrial oxidative phosphorylation and fatty acid oxidation in 3T3-L1 adipocytes. These findings provide a crucial framework for the engineering of novel structure-based variants of FGF21 with improved stability and biological activity to treat metabolic disorders.

## Why it matters

Metabolic diseases, such as obesity and diabetes, are serious global health concerns. Fibroblast growth factor 21 (FGF21) is a key metabolic regulator mediating glucose and fatty acid metabolism. Thus, FGF21 has significant potential as a therapeutic agent for the treatment of diabetes and obesity. However, limited research is available on its structure and stability. Herein, we characterize the structures and stability of a recombinant FGF21 using biophysical methods. The results of this study provide valuable insights for the development of potent FGF21-based therapeutic agents to combat dilapidating metabolic diseases. Additionally, this study paves a way for more in-depth structural studies of FGF21 in the future and facilitates a better understanding of FGF21’s mode of action.

## Introduction

Fibroblast growth factors (FGFs) are a group of 22 proteins in the human body that regulate various critical biological processes, including angiogenesis and wound healing (1). Endocrine FGFs are members of the FGF19 subfamily that share the ability to function similarly to hormones and mediate vital biological regulations, such as bile acid synthesis, phosphate regulations, and glucose and fatty acid metabolism (2). FGF21 is an endocrine FGF expressed in hepatocytes and adipocytes, meditating lipid and glucose metabolism (1,3). Unlike paracrine FGFs, FGF21 has a low binding affinity to heparin sulfate (HS) (2), facilitating it to traverse throughout the body via the bloodstream and function like a hormone (4). FGF21 expression in humans and mouse models is complex and influenced by many factors, including exercise, diet (fasting or feeding with low-calorie or ketogenic diets), and other metabolic conditions (5). Diets high in sucrose and fat are also known to increase FGF21 expression in the liver and pancreatic islets (5).

The precursor of FGF21 comprises 209 residues and is processed into an 181-residue mature protein (∼19,400 Da) through the cleavage of the N-terminal signal peptide (6). FGF21 has an unstructured 40-residue C-terminus and a 13-residue N-terminal region, flanking the core domain (7). This core structure consists of a noncanonical β-trefoil motif with 11 β strands, where an 18-residue proline-rich disordered loop replaces the β11 strand (7). This β11 strand contains a conserved glycine box motif (GXXXXGXX(T/S)) essential for HS binding through tight hydrogen bonds in paracrine FGFs (8). The absence of the β11 strand and the glycine box motif in FGF21 results in the protein’s reduced affinity for HS (7). Additionally, the HS binding region of FGF21 is more negatively charged than other paracrine FGFs, which repels the binding to the sulfate groups on HS and lowers the overall affinity for HS (7). Moreover, FGF21’s noncanonical core structure exhibits inherent flexibility, leading to conformational instability. Specifically, the proline-rich disordered loop in β11 influences the folding of the nearby β2-β3 hairpin, creating inherent dynamic folding within this region and negatively impacting the protein’s overall stability. FGF21 is also prone to proteolytic cleavage, resulting in FGF21’s low in vivo half-life (0.5–2 h) (7,9). Both the N-terminal and C-terminal regions of FGF21 are crucial for binding to receptors. The N-terminus interacts with FGF receptors (FGFRs) (1). The C-terminus serves as an anchor for β-klotho (KLB) (1) but is particularly susceptible to proteolytic cleavage in vivo, which affects the protein’s binding to KLB (10,11).

FGF21 functions are diverse. In adipocytes, FGF21 increases energy expenditure by promoting glucose utilization and the expression of the glucose transporter-1, a transmembrane glucose transport channel (12). FGF21 also reduces blood glucose levels in an insulin-independent manner (12) and facilitates lipid uptake into brown adipocytes (13). In the liver, FGF21 has been demonstrated to reduce lipogenesis (4,14), increase insulin sensitivity and fatty acid oxidation (15,16,17), and prevent ectopic lipid accumulation (18). Elevated FGF21 serum levels are found in patients with fatty liver diseases, such as nonalcoholic fatty liver diseases and alcoholic fatty liver diseases (15). The increase in serum FGF21 serves as a protective response to liver lipotoxicity, enhancing fatty acid β-oxidation and reducing lipogenesis to improve liver function in these metabolic diseases (15,19). Consequently, FGF21 has emerged as a promising therapeutic for treating metabolic diseases (9), given its beneficial effects on metabolic regulation (15). Diseases such as obesity, type 2 diabetes, and nonalcoholic steatohepatitis (now called metabolic dysfunction-associated steatohepatitis (20)) have been treated with FGF21 analogs, with promising results (19,21,22).

Despite the growing body of research on FGF21’s biological functions since its initial discovery in the 2000s (23), there is a shortage of studies on its biophysical characterizations and stability. To address this knowledge gap, our study aims to investigate FGF21’s structure and function by studying the structural elements and stability of recombinant FGF21 (rFGF21) using biophysical methods. Additionally, the production of rFGF21 is often challenging, with issues including insolubility and low bioactivity (24). Herein, we report the overexpression and purification of soluble rFGF21 using an *Escherichia coli* (*E. coli*) expression system (*Rosetta-gami*), which can enhance the protein’s solubility and bioactivity (25). Furthermore, we explore the biological activities of rFGF21 on NIH/3T3 fibroblasts and 3T3-L1 adipocytes to better understand the structure-function relationship of the protein. By combining biophysical characterization techniques with biochemical methods, these new findings expand our understanding of FGF21’s impact on mitochondrial functions and its involvement in treating metabolic diseases. Thus, the results also provide a crucial foundation for developing novel, hyper-stable FGF21 variants with enhanced biological activities for treating metabolic diseases through structure-based protein engineering.

## Materials and methods

The required materials and compositions of different buffers and media are provided in Fig. S1.

### Construction and purification of rFGF1 and rFGF21

The genes encoding truncated human FGF1 (residues 16–155) (rFGF1) and mature human FGF21 (residues 29–209) (rFGF21) were cloned into pET21a+ vectors containing an ampicillin resistance gene (Genscript, Piscataway, New Jersey). FGF21 was expressed in *Rosetta-gami* cells, while rFGF1 was expressed in BL21 Star (DE3) cells. The cells were cultured in Miller's Luria Broth medium to an optical density of 0.6–0.8 (at 600 nm), induced with 1 mM IPTG, and incubated for 5 h at 37°C with shaking at 240 rpm (26). The cells were harvested, resuspended in the working buffer, and sonicated on ice using a Sonifier SFX150 (Branson Ultrasonics, Danbury, Connecticut) (10-s on/off pulses for 15 min, repeated twice with a 15-min break). The lysates were centrifuged at 19,000 rpm for 25 min using Avanti J-25 Centrifuge (Beckman Coulter, Brea, California). rFGF21 supernatant was passed over a nickel Sepharose column equilibrated with the working buffer at 1 mm per minute. rFGF21 was eluted using a stepwise IMD gradient (20–500 mM). rFGF1 supernatant was passed over a heparin Sepharose column, equilibrated with the working buffer. rFGF1 was eluted using a stepwise NaCl gradient (100–1500 mM). The purification process was monitored by absorption at 280 nm. Proteins were collected and run on sodium dodecyl sulfate-polyacrylamide gel electrophoresis (SDS-PAGE; 15%, 200 V, 50 min), stained with Coomassie brilliant blue. Protein concentrations were determined using the Bradford method and NanoDrop Microvolume Spectrophotometers (Thermo Fisher Scientific, Waltham, Massachusetts) (27,28).

### Western blot

Purified his-tagged rFGF21 (31.4 *μ*M) was run on SDS-PAGE (15%, 200 V, 50 min). The resulting gel was transferred to a 0.2 *μ*m nitrocellulose membrane in Towbin (25 mM Tris, 192 mM glycine [pH 8.3], 20% v/v methanol) at 150 V, 80 mA, 120 min. The membrane was blocked in 5% skim milk in TBS-T solution (20 mM Tris, 150 mM NaCl, 0.1% w/v Tween 20) for 30 min, rinsed, then incubated in 0.2% BSA in TBS-T with added alkaline phosphatase anti-6X his antibody at room temperature overnight, and washed with NBT/BCIP solution the following day to visualize the his-tagged bands (26).

### Mass spectrometry

Proteins (31.4 *μ*M) were subjected to intact liquid chromatography-electrospray ionization (ESI)-mass spectrometry (MS) to determine their molecular weights through the service of the University of Arkansas Statewide Mass Spectrometry Center (26).

### Circular dichroism and fluorescence spectrophotometry

Proteins (31.4 *μ*M) in 10 mM phosphate buffer and 100 mM NaCl were analyzed using a J-1500 Spectrophotometer (JASCO, Tokyo, Japan) in a 0.1 mm quartz cell with the wavelength set to 190–250 nm at 25°C and a 20 nm/min scanning speed. Ten scans were averaged, analyzed, and normalized (26).

Fluorescence spectra were obtained using an F-2500 Fluorescence Spectrophotometer (Hitachi High-Tech, San Diego, California) with excitation at 280 nm and emission measured from 300 to 450 nm. Background noise was eliminated by subtracting the buffer scans from the data. Ten scans were averaged, analyzed, and normalized (26).

### ANS binding assay

Proteins (31.4 *μ*M) in a 10 mm quartz cuvette were titrated with 8-anilino-1-napthalenesulfonic acid (ANS) stock (20 mM) by the addition of 10 μM increments. After mixing and incubating at 25°C, fluorescence was measured using the F-2500 Spectrophotometer (Hitachi High-Tech) with excitation at 380 nm and emission from 450–600 nm (2.5 nm slit width). The data at 520 nm were analyzed (26).

### Limited trypsin digestion assay

Control groups included tubes with only protein (tube 1) and only trypsin (tube 10), both containing 200 *μ*L of solution. For the concentration-dependent assay, eight tubes (tubes 2–9) containing 31.4 *μ*M of protein each were incubated with different bovine trypsin concentrations (shown in results and discussion) at 37°C for 40 min. The incubation times for tubes 1 and 10 were 0 min. For the time-dependent assay, eight tubes (tubes 2–9) containing 31.4 *μ*M of protein and 0.0005 mg/mL trypsin in the working buffer (total volume of 200 *μ*L each) were incubated at 37 °C at various time intervals (shown in results and discussion). The incubation times of tubes 1 and 10 were 0 min. 10% trichloroacetic acid was added to stop the reaction. Samples were run on SDS-PAGE (15%, 50 min, 200 V) and stained. UN-ScanIT densiometric software (Silk Scientific, Orem, Utah) analyzed the gel, and protein digestion was quantified as the percentage of undigested protein compared to tube 1 except for tube 10 (26).

### Differential scanning calorimetry

Proteins (31.4 *μ*M) were analyzed using MicroCal PEAQ-DSC (Malvern Panalytical, Almelo, the Netherlands) with a temperature range from 25°C to 80°C and a 1°C ramping rate. Three buffer scans were conducted and subtracted from the sample to eliminate noise. Specific heat capacity and melting temeprature (*T*_*m*_) were calculated using MicroCal PEAQ-DSC software and plotted (26).

### Isothermal titration calorimetry

Binding interactions of proteins (31.4 *μ*M, degassed) and HS were monitored using a MicroCal iTC200 Micro Calorimeter (Malvern Panalytical) monitored the binding process of proteins (31.4 *μ*M, degassed). HS (251.2 *μ*M) in the syringe was titrated into proteins in a 1:8 protein:ligand ratio. The injection volume was set at 2 *μ*L, with 20 titrations at 25°C. Data were analyzed using the accompanying Origin software (26).

### Equilibrium unfolding using chemical denaturant

rFGF1 (31.4 *μ*M in 1.5 mL working buffer) was titrated with 8 M urea using the J-1500 Spectrophotometer with automatic scanning titration. The excitation wavelength was set at 280 nm, with an emission range from 300 to 450 nm. The fluorescence intensity at 305 and 350 nm was analyzed, the 305/350 nm ratio was calculated, and the chemical transition midpoint (*C*_*m*_) was determined and plotted as reported elsewhere (26,29).

rFGF21 (31.4 *μ*M in 1.5 mL working buffer) was mixed with 4 *μ*L SYPRO Orange stock (5000×) and titrated with 8 M urea by adding 20 *μ*L increments until fluorescence intensity decreased, monitored by F-2500 Spectrophotometer in a 1 cm quartz cell as done elsewhere (26,30). The excitation wavelength was 491 nm, and the emission range was 500–650 nm. Fluorescence values at a 586 nm wavelength were analyzed, and the *C*_*m*_ was determined. Data after 100% protein unfolded were not plotted, as noted elsewhere (30,31).

### Cell proliferation activity

NIH-3T3 fibroblast cells were maintained at 37°C, 5% CO_2_, in DMEM containing 10% BCS and 1% penicillin-streptomycin. When cells reached 70% confluence, they were trypsinized and seeded (10,000 cells/well) in a 96-well plate. Cells were treated with varying concentrations of rFGF21 or rFGF1, and cell counts were quantified after 24 h using Hoechst 33342 dye and a BioTek Cytation 5 (Agilent, Santa Clara, California).

### Seahorse assay

3T3-L1 adipocytes were grown in DMEM containing 10% BCS at 37°C and 5% CO_2_ until 70% confluence and trypsinized. In the Seahorse 96-well plate, 5000 cells were seeded in each well in DMEM with 10% BCS for 3 days; differentiated in DMEM with 10% FBS, 0.5 mM IBMX, 1 *μ*M dexamethasone, and 10 *μ*g/mL insulin for 3 days; continued in DMEM with 10% FBS and 10 *μ*g/mL insulin for 3 more days, and finished in DMEM with 10% FBS for 3 final days (adapted from (32)). For the ATP production assay, cells were treated with 3.125 nM (∼50 ng/mL) of proteins for 15 h, washed, prepared for XF ATP Real-Time rate assay (Agilent), and analyzed by Seahorse XF Pro Analyzer (Agilent). For the palmitate oxidation assay, cells were starved in DMEM only and treated with 0.625 nM (∼10 ng/mL) of products for 15 h, washed, and prepared for the XF Palmitate Oxidation Stress Test assay (Agilent), followed by the addition of palmitate:BSA stock (1 mM) (Agilent) (final concentration of 167 *μ*M) for 15 min before being analyzed by Seahorse XF Pro Analyzer. Data were analyzed using Seahorse Analytics and plotted.

### Data analysis

Experiments for structural characterizations were performed with three independent replicates (*n* = 3). For cell-based in vitro studies, at least five wells (*n* = 5) were used per condition, as specified in the figure legends. Data were analyzed using one-way ANOVA followed by the Bonferroni post hoc test for multiple group comparisons. Differences between any two groups were analyzed using unpaired two-tailed Student’s *t*-tests. Results are expressed as mean ± SD.

## Results and discussion

### Purification and authentication of soluble rFGF21

**Figure 1:**
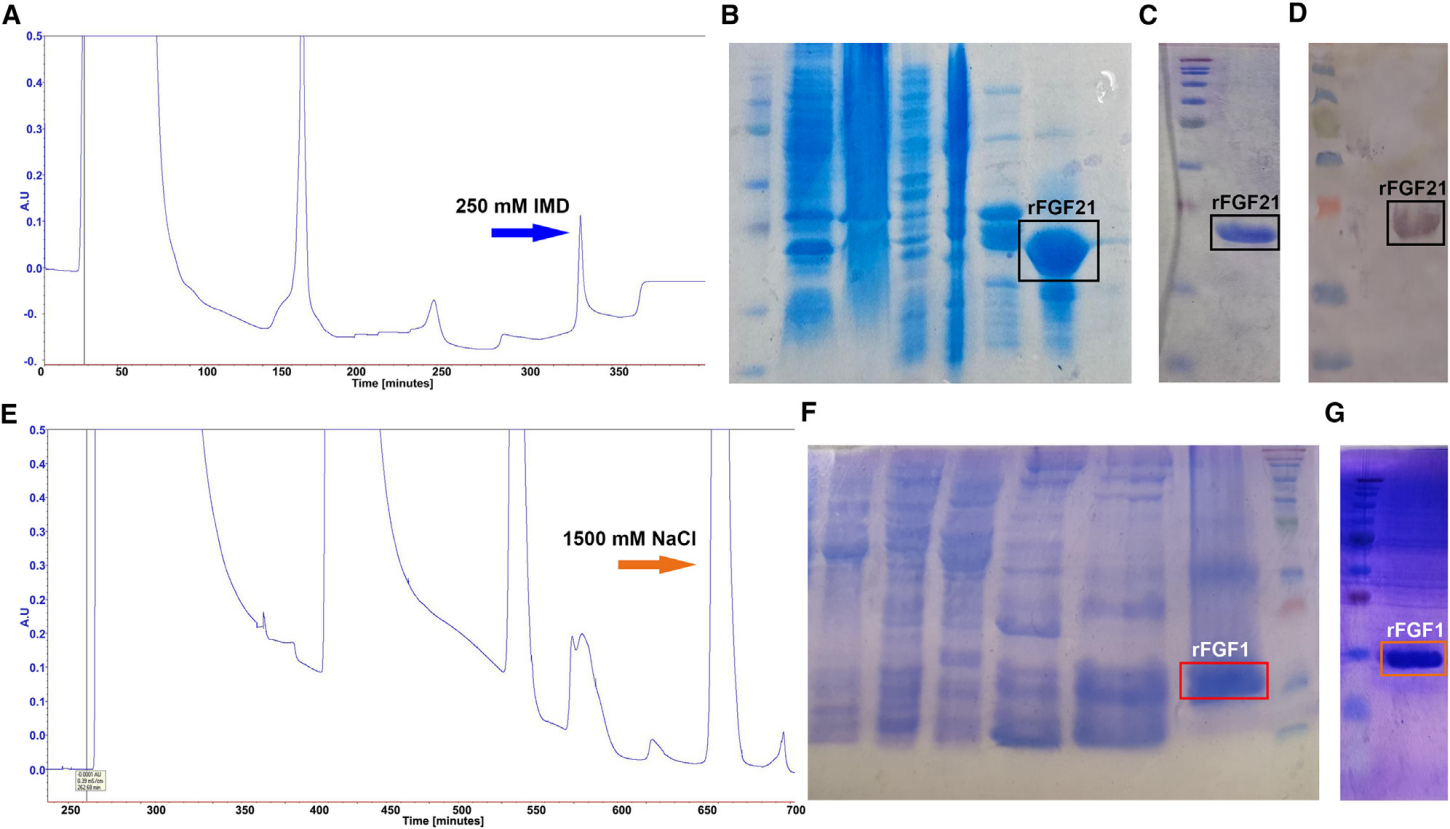
Purification of rFGF21 and rFGF1. rFGF21 is expressed in *Rosetta-gami* and purified using Ni^2+^ Sepharose column (*A*). The protein is eluted at 250 nM IMD and confirmed through SDS-PAGE (*B*). The product purity is confirmed (*C*). His-tagged rFGF21 is confirmed via western blot using the anti-His antibody (*D*). rFGF1 is expressed in *BL21 Star*, purified using the Heparin Sepharose column (*E*). The protein was eluted at 1500 mM NaCl and confirmed with SDS-PAGE (*F*). The product purity is confirmed through SDS-PAGE (*G*).

The production of human FGF21 in *E. coli* bacterial hosts is challenging due to various problems such as inclusion body formation, low yield of soluble protein, and decreased bioactivity (24,25,32) (Figs. S2 *A*, S3 *A*, and S4 *A*). Protein that forms inclusion bodies requires denaturation and renaturation processes, which further lower the yield of bioactive samples (24). Another challenge is the presence of one disulfide bridge in FGF21, which is difficult to form in conventional BL21 (DE3) *E. coli* strains due to their reducing cytoplasm (33,34). Herein, we report the overexpression of 6X his-tagged rFGF21 (∼20.364 kDa) in the *Rosetta-gami* bacterial strain, which has an oxidizing cytoplasm conducive to forming a disulfide bond within FGF21 (33). Similar expression strategies have been shown to produce a higher yield and purity of rFGF21 (35,36). Indeed, the his-tagged rFGF21 expressed in the *Rosetta-gami* cell line is soluble after IPTG induction (Figs. S2 *A*, S3 *B*, and S4 *B*) and purified using the Ni^2+^ Sepharose column (Fig. 1 *A*). SDS-PAGE analysis after purification confirms that rFGF21 elutes at 250 mM IMD (Fig. 1 *B*). After concentration and buffer exchange, rFGF21 appears pure on the SDS-PAGE gel (Fig. 1 *C*), with a concentration of 1.3 mg/mL and a yield of 8.5 mg/L of culture. Paracrine FGFs, like human FGF1 (rFGF1), do not suffer from expression or inclusion body problems. rFGF1 is expressed in BL21 Star (DE3) (Figs. S2 *B* and S3 C) and purified using a heparin Sepharose column due to its high affinity to heparin (Fig. 1 *E*). rFGF1 elutes at a high-salt fraction (1500 mM NaCl), yielding a high concentration (6.55 mg/mL) and total yield (24.6 mg/L of cell culture) (Fig. 1, *F* and *G*).

**Figure 2:**
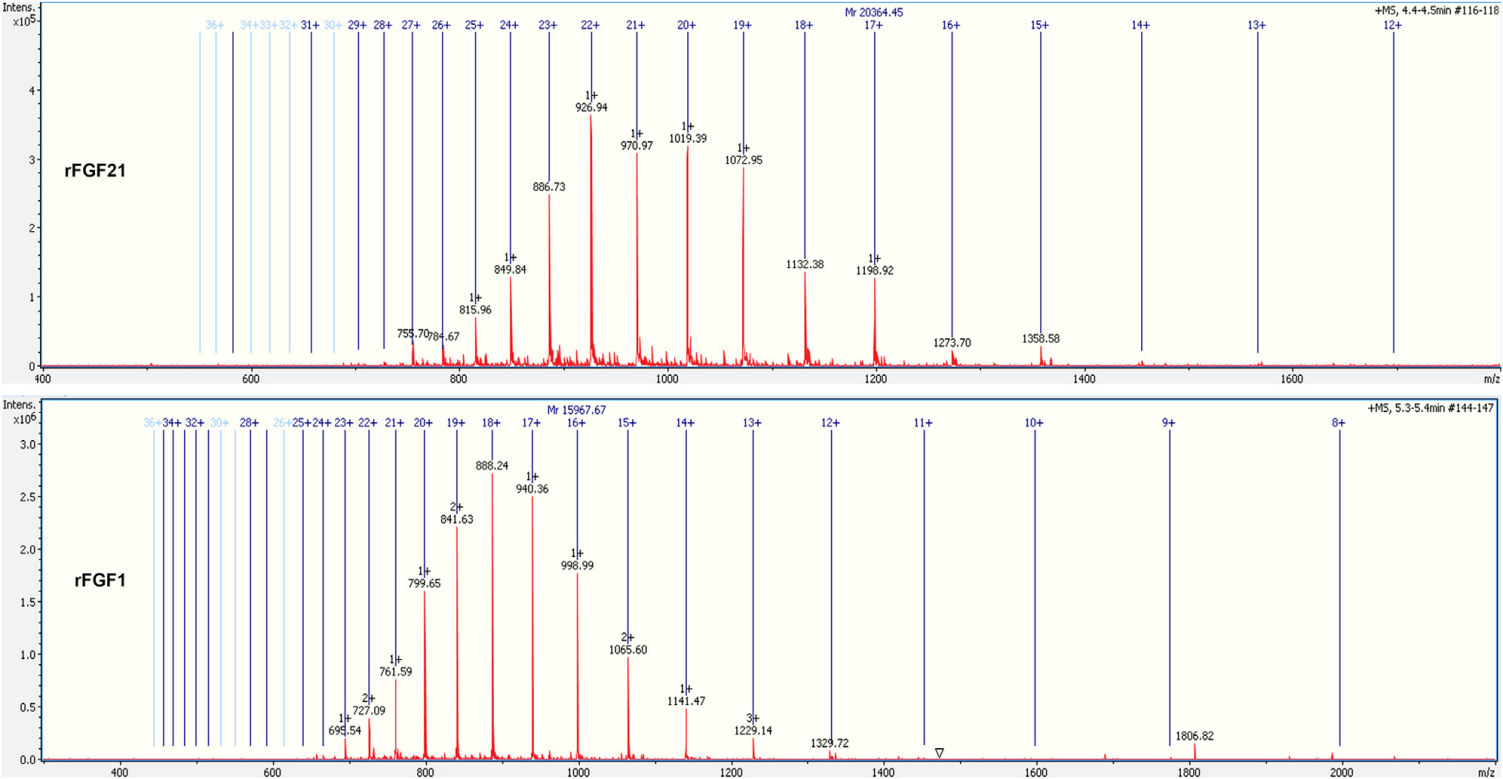
The molecular weights of rFGF21 and rFGF1 using mass spectrometry. Purified samples are authenticated using intact liquid chromatography (LC)-MS. The top shows rFGF21’s molecular weight (∼ 20.364 kDa), matching the rFGF21 design. The bottom shows rFGF1’s molecular weight (∼15.9 kDa), matching the rFGF1 design (adapted from (26)).

To authenticate the purified products, we employed both western blot and MS methods. Since the rFGF21 design is his tagged, we performed a western blot using an anti-his antibody, which revealed a band between 15 and 30 kDa (Fig. 1 *D*), matching rFGF21’s molecular weight and confirming the presence of a his-tagged rFGF21 in purified products. Purified products are also analyzed by ESI-MS to determine their molecular weights. The product of Ni^2+^ Sepharose purification has a molecular weight of 20.364 kDa, matching the expected molecular weight of rFGF21. Similarly, the product of heparin Sepharose purification has a molecular mass of 15.967 kDa, consistent with rFGF1’s theoretical molecular weight (Fig. 2). Both the western blot and the MS results verify that the products are correctly identified as rFGF21 and rFGF1, confirming the successful expression and purification of both proteins. These findings suggest that rFGF21 can be effectively produced in the *Rosetta-gami* bacterial line, resulting in soluble and pure proteins with high yields that can meet experiment demands.

### Biophysical characterization of rFGF21

**Figure 3:**
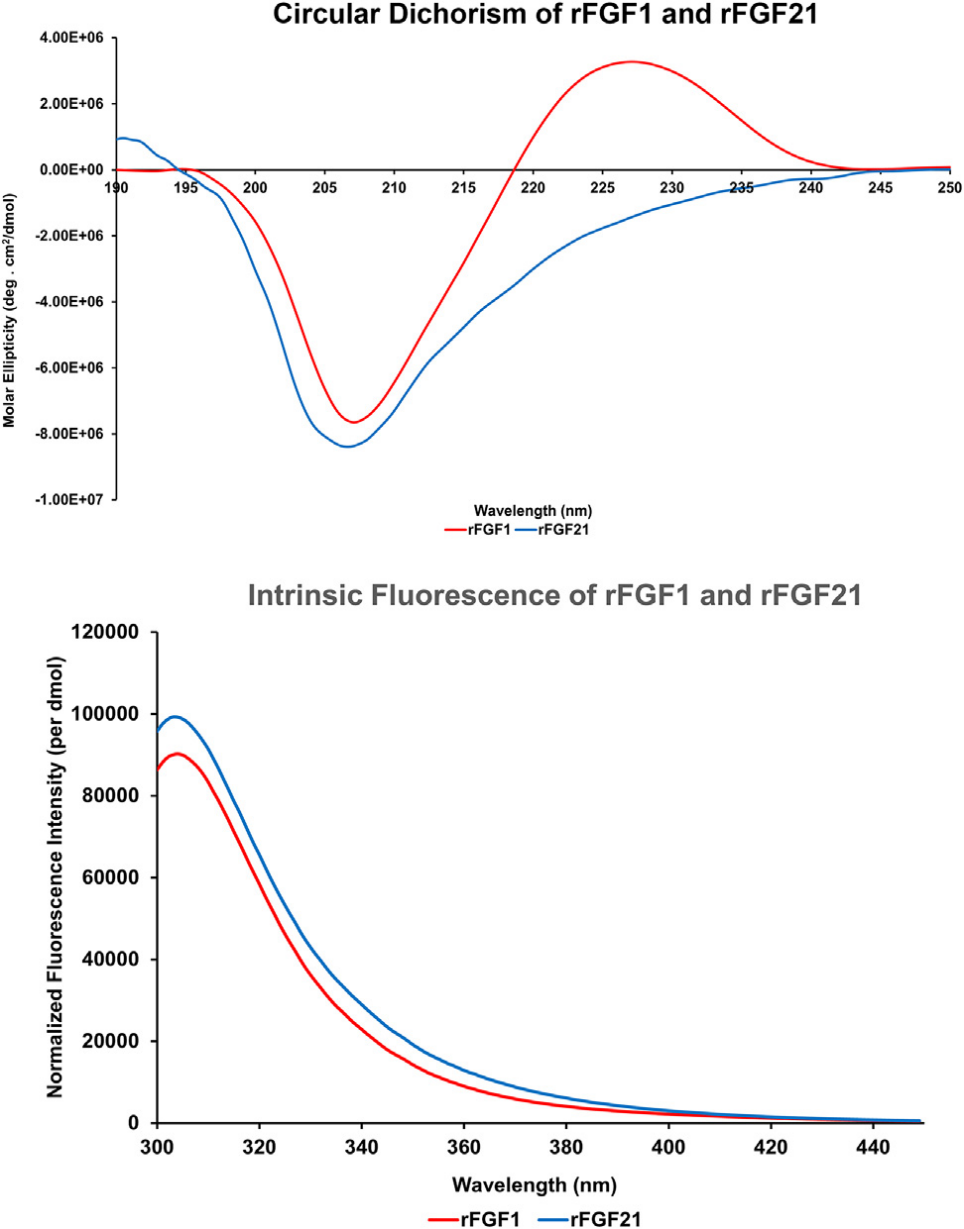
Far-UV circular dichroism and intrinsic fluorescence spectra of rFGF21 and rFGF1. The top panel displays the circular dichroism spectra of products in molar ellipticity. rFGF1 has one negative peak at 208 nm and a positive peak at 228 nm. rFGF21 has one negative peak at 208 nm. The bottom panel shows the products’ intrinsic fluorescence at 308 nm. rFGF21 is in blue and rFGF1 is in red.

Although there has been considerable research into FGF21’s biological functions, detailed biophysical studies of rFGF21 are lacking. Herein, we investigate the structural elements and stability of endocrine rFGF21 and compare them to the paracrine rFGF1 to highlight the differences between endocrine and paracrine FGFs in terms of structures and stability. The far-UV circular dichroism spectrum of rFGF21 shows one negative peak around 208 nm, indicative of a β sheet structure from the conserved β-trefoil core common to all FGFs (7). The rFGF1 spectrum also displays a negative trough around 208 nm, consistent with a β sheet core structure (37). However, rFGF21 lacks the positive peak at 228 nm observed in rFGF1, which is attributed to the aromatic side chains in rFGF1 (Fig. 3). Mature rFGF21 possesses all aromatic residues except tryptophan, while rFGF1 has one vital tryptophan residue along with phenylalanine and tyrosine (37). The absence of said tryptophan residue in rFGF21 could contribute to the lack of a positive peak at 228 nm.

The intrinsic fluorescence spectra of both proteins showed a tyrosine fluorescence emission at 308 nm. rFGF1 contains eight tyrosine residues and one tryptophan residue, which has its intrinsic fluorescence at 350 nm peak. This tryptophan is often quenched in the native conformation of rFGF1 and can be observed upon unfolding (37) (Fig. S5 *A*). In contrast, rFGF21 lacks tryptophan and only displays tyrosine emission at 308 nm (Fig. S5 *B*). These results indicate that both rFGF21 and rFGF1 are correctly folded and likely biologically active.

**Figure 4:**
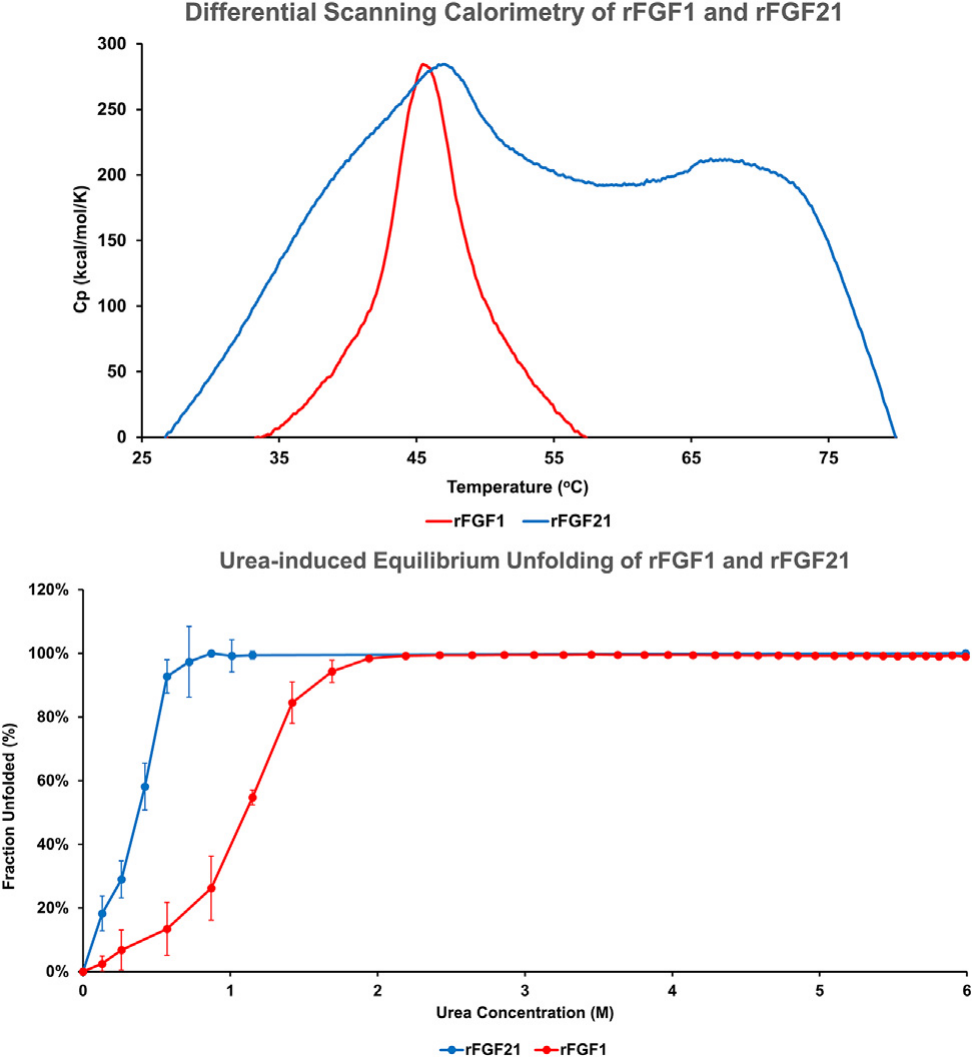
Thermal and chemical stability of rFGF21 and rFGF1. The product’s thermal stability using differential scanning calorimetry (DSC) is shown on the top. rFGF1 showed a *T*_*m*_ at 45.47°C (±0.1°C). rFGF21 showed a *T*_*m*_ at 46.7°C (±0.1°C) but has a second *T*_*m*_ at 67.15°C (±0.1°C) and a broad peak. The product’s stability in 8 M urea is shown on the bottom. The *C*_*m*_ of FGF21 is 0.4 M, and that of rFGF1 is 1.2 M. rFGF21 is in blue and rFGF1 is in red.

The melting temperatures (*T*_*m*_) of rFGF21 and rFGF1 were determined using differential scanning calorimetry. rFGF1 shows a *T*_*m*_ of around 45.5°C (±0.1°C), consistent with previous reports on the protein’s low thermal stability (37) (Fig. 4). rFGF21 exhibits a *T*_*m*_ of around 46.7°C (±0.1°C) and a second *T*_*m*_ at 67.1°C (±0.1°C) with a broad peak, suggesting that FGF21 may undergo multiple thermal-unfolding states (38) (Fig. 4). Interestingly, it is reported that the apparent *T*_*m*_ of rFGF21 is at 37°C, at which point the protein unfolds, and aggregation occurs (38), further corroborating rFGF21’s low thermal stability, even in comparison to the thermally unstable rFGF1.

Similarly, urea-induced unfolding curves (pH 7.2) of both proteins showed that rFGF21 is more prone to unfolding than rFGF1, which itself is relatively unstable in urea (38). The equilibrium unfolding data revealed that rFGF21 has a low *C*_*m*_ of 0.4 M, whereas rFGF1 is 1.2 M (Fig. 4). Overall, the results confirm that rFGF21 is thermally and chemically unstable.

**Figure 5:**
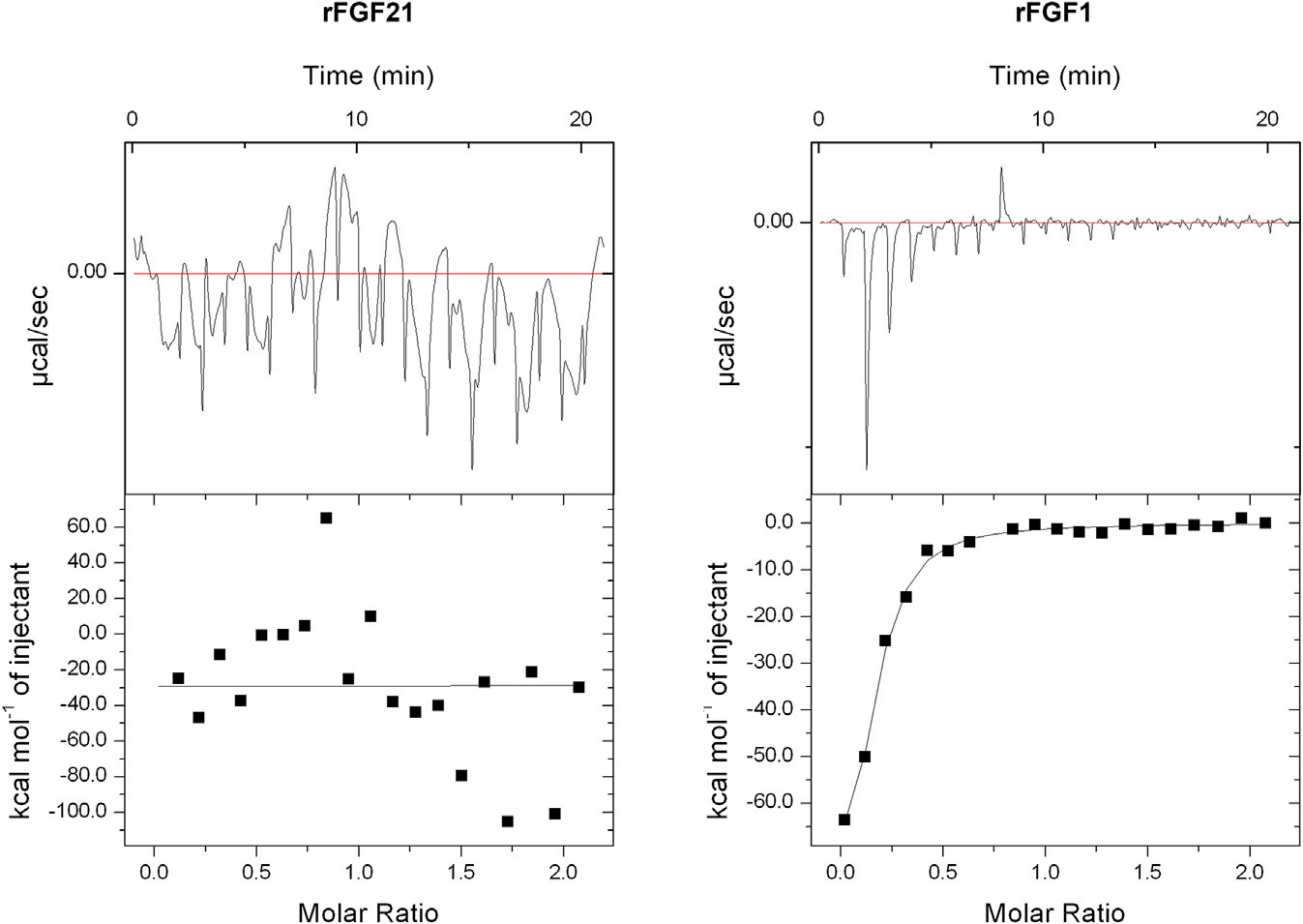
Isothermogram of rFGF21 and rFGF1. The figure shows the heparin affinity of rFGF21 (*left*) and rFGF1 (*right*). rFGF21 does not display any heparin binding affinity. rFGF1 showed binding affinity to heparin, K_D_ = ∼2.9 *μ*M (±1.3 *μ*M). Results are expressed as mean ± SD.

Isothermal titration calorimetry is a versatile technique used to measure the binding affinity of protein-ligand interactions (39) and was employed to assess the binding affinities of rFGF21 and rFGF1 for low-molecular-weight HS (∼ 3 kDa). Paracrine FGFs like rFGF1 exhibit a high affinity to HS, while endocrine FGFs like rFGF21 do not (32,39). The isothermograms reveal that rFGF21 has no affinity for HS, while rFGF1 demonstrates a notable binding affinity for HS (K_D_ = ∼2.9 ± 1.3 *μ*M) (Fig. 5).

**Figure 6:**
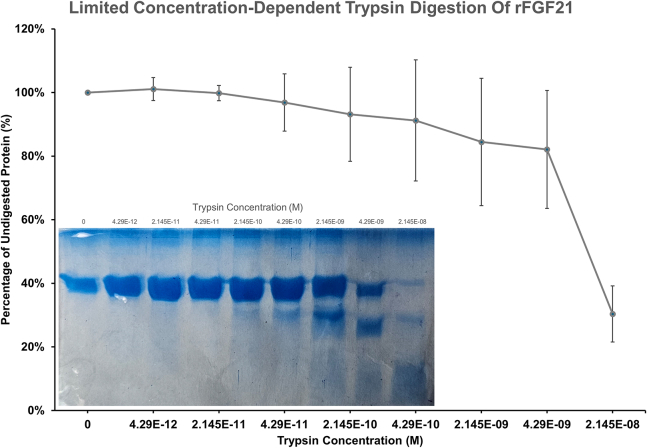
Limited concentration-dependent trypsin digestion assay of rFGF21. The figure shows rFGF21 digestion using small concentrations (0–2.15 × 10^−8^ M, equivalent to 0.0005 mg/mL) at 37°C for 40 min (*inset*). 0.0005 mg/mL trypsin concentration can digest rFGF21 easily, with 30% of rFGF21 remaining undigested. This concentration was chosen to digest both rFGF21 and rFGF1 in a time-dependent digestion assay.

**Figure 7:**
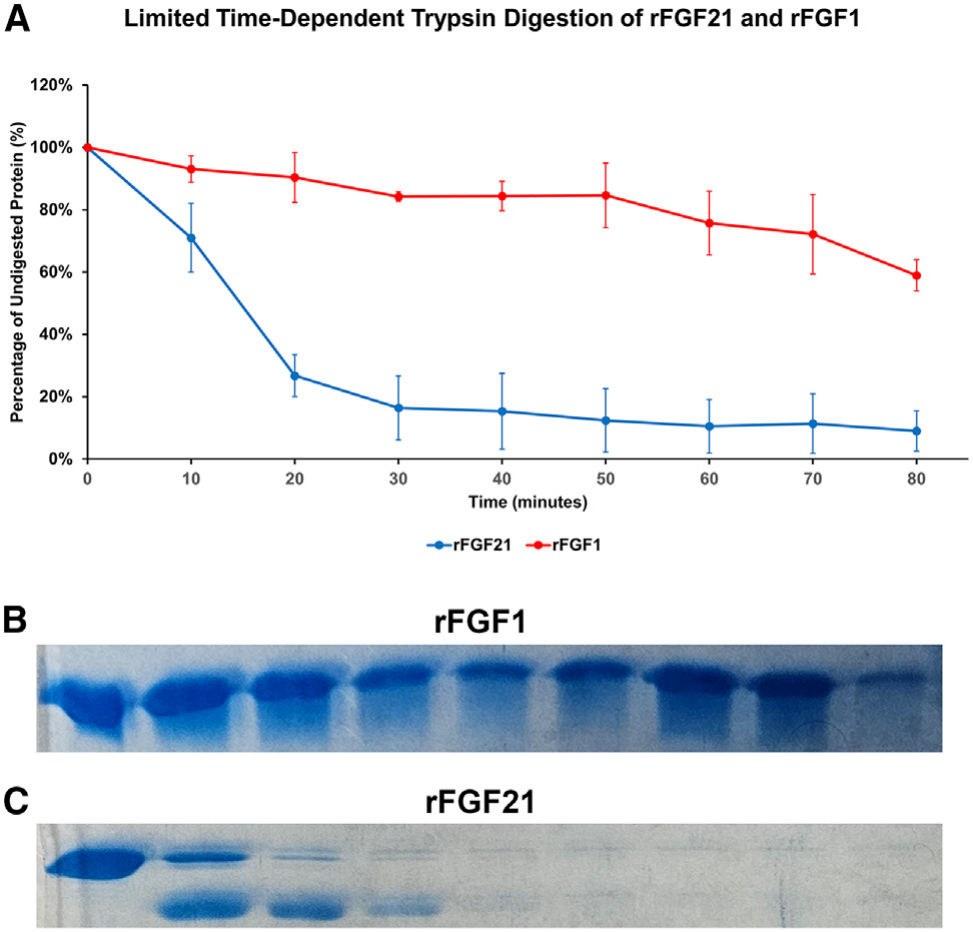
Limited time-dependent trypsin digestion assay of rFGF21 and rFGF1. The figure shows the product’s trypsin digestion using 2.15 × 10^−8^ M (0.0005 mg/mL) trypsin concentration at 37°C for 0–80 min (*B* and *C*, respectively). rFGF1 is steadily digested by trypsin from 0 to 80 min with ∼59% (±5%) of undigested protein after 80 min (*A*). rFGF21 was digested rapidly with only ∼9% (±6%) of undigested protein after 80 min (*A*). rFGF21 is in blue and rFGF1 is in red. Results are expressed as mean ± SD.

rFGF1 contains 11 lysines and six arginines (39), while rFGF21 has 10 arginines and four lysines. Limited trypsin digestion is used to assess both proteins’ resistance to trypsin digestion and the structural flexibility of their backbones. In a concentration-dependent trypsin assay, rFGF21 is shown to be susceptible to trypsin digestion at low trypsin concentrations (0–2.145 × 10^−8^ M, equivalent to 0.0005 mg/mL) after 40 min at 37°C (Fig. 6, *inset*). At a 0.0005 mg/mL trypsin concentration, 30% of rFGF21 remained undigested (Fig. 6). Therefore, this trypsin concentration is then used in a time-dependent trypsin assay from 0 to 80 min for both rFGF1 and rFGF21 to compare their structural flexibility. The result shows that rFGF21 is digested rapidly, with around 9% (±6%) of the protein remaining undigested after 80 min (Fig. 7). Under similar conditions, rFGF1 is notably more resistant to trypsin digestion than rFGF21, with 59% (±5%) of the protein remaining undigested after 80 min (Fig. 7). These results suggest that rFGF21 is more prone to trypsin digestion, even at low concentrations, and may possess a more structurally flexible backbone than rFGF1, allowing trypsin to access and digest rFGF21 easily.

**Figure 8:**
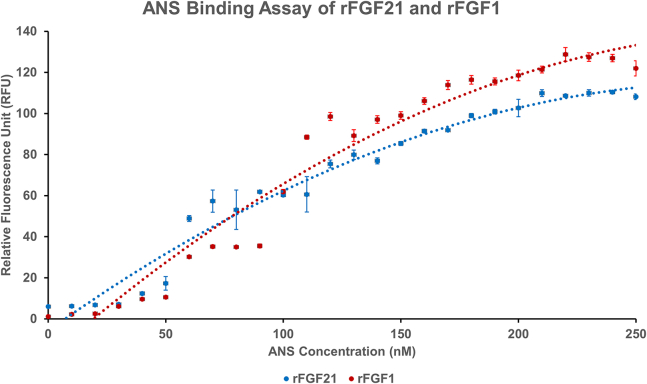
ANS binding curves of rFGF21 and rFGF1. The figure shows the binding between the proteins to ANS. rFGF21-ANS binding fluorescence at 520 nm peaks at 240 nM of ANS (110.4 ± 0.3 RFU) and rFGF1-ANS at 220 nM ANS (128.7 ± 3.4 RFU). rFGF21 requires more ANS to reach maximum fluorescence than rFGF1 and may have more solvent-exposed hydrophobic pockets. rFGF21 is in blue and rFGF1 is in red. Results are expressed as mean ± SD.

The structural flexibility of rFGF21 is further monitored by an ANS binding assay. ANS is a nonpolar dye that detects solvent-exposed hydrophobic pockets within proteins. These pockets are typically buried within the protein core. Increased ANS fluorescence indicates a greater amount of solvent-exposed hydrophobic pockets (40). ANS binding curves for rFGF21 and rFGF1 are similar, with some notable differences. rFGF21 reached maximum relative fluorescence at 240 nM of ANS (110.4 ± 0.3 relative fluorescence unit [RFU]), higher than rFGF1, which reached a maximum with 220 nM ANS (128.7 ± 3.4 RFU) (Fig. 8). Since rFGF21 requires more ANS than rFGF1 to reach maximum fluorescence, rFGF21 has more solvent-exposed hydrophobic pockets that can access ANS for binding than rFGF1 and may be more structurally flexible than rFGF1. The ANS assay, together with the trypsin digestion data, suggests that rFGF21 is more dynamically flexible than rFGF1.

### Functional characterization of rFGF21

**Figure 9:**
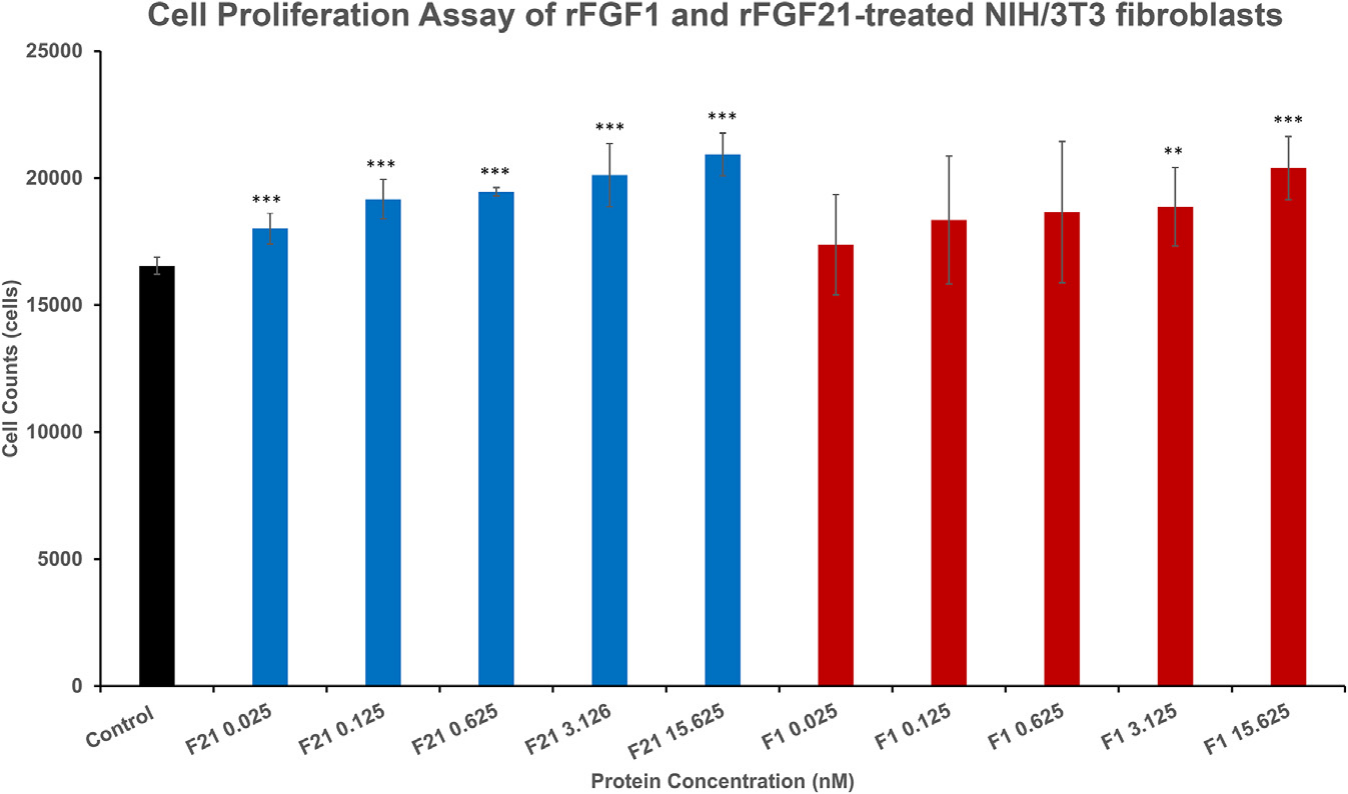
Cell proliferation activities of rFGF21 and rFGF1 on NIH/3T3 cells. The figure shows the proliferation of protein-treated NIH/3T3 fibroblasts at various concentrations (0.025–15.625 nM). rFGF1-treated fibroblasts (at 3.125 and 15.625 nM) have statistically higher cell numbers than the control. rFGF21-treated cells also have statistically higher cell numbers than the control. rFGF21 is in blue and rFGF1 is in red. ∗∗*p* ≤ 0.01, ∗∗∗*p* ≤ 0.001, and α∗-value = 0.005.

rFGF1 is a mitogenic FGF with potent cell proliferative activities on fibroblasts (37). In contrast, Kharitonenkov et al. reported that rFGF21 does not exhibit proliferative effects on NIH/3T3 fibroblasts (32). Our data show that rFGF1 induces NIH/3T3 cells to grow, particularly at 3.125 and 15.625 nM concentrations, where cell counts were significantly higher than the control group (Fig. 9). Surprisingly, rFGF21 also exhibits mitogenic effects on NIH/3T3 cells, with cell numbers at all rFGF21 concentrations being significantly higher than the control (Fig. 9). This result contradicted earlier reports that rFGF21 lacks mitogenic activity (32) but is consistent with more recent studies showing that rFGF21 can promote the proliferation of NIH/3T3 cells (36,41). The mitogenic activity of FGF21 may be attributed to its affinity for FGFR1c, its preferred receptor (34,42,43). FGFR1c regulates FGF21’s metabolic activities in target tissues like adipocytes and plays critical roles in FGF21’s biological functions (44,45). As FGFR1c is expressed in NIH/3T3 fibroblasts and can stimulate their proliferation (43,46), rFGF21 likely interacts with this receptor in fibroblasts to promote cell proliferation.

**Figure 10:**
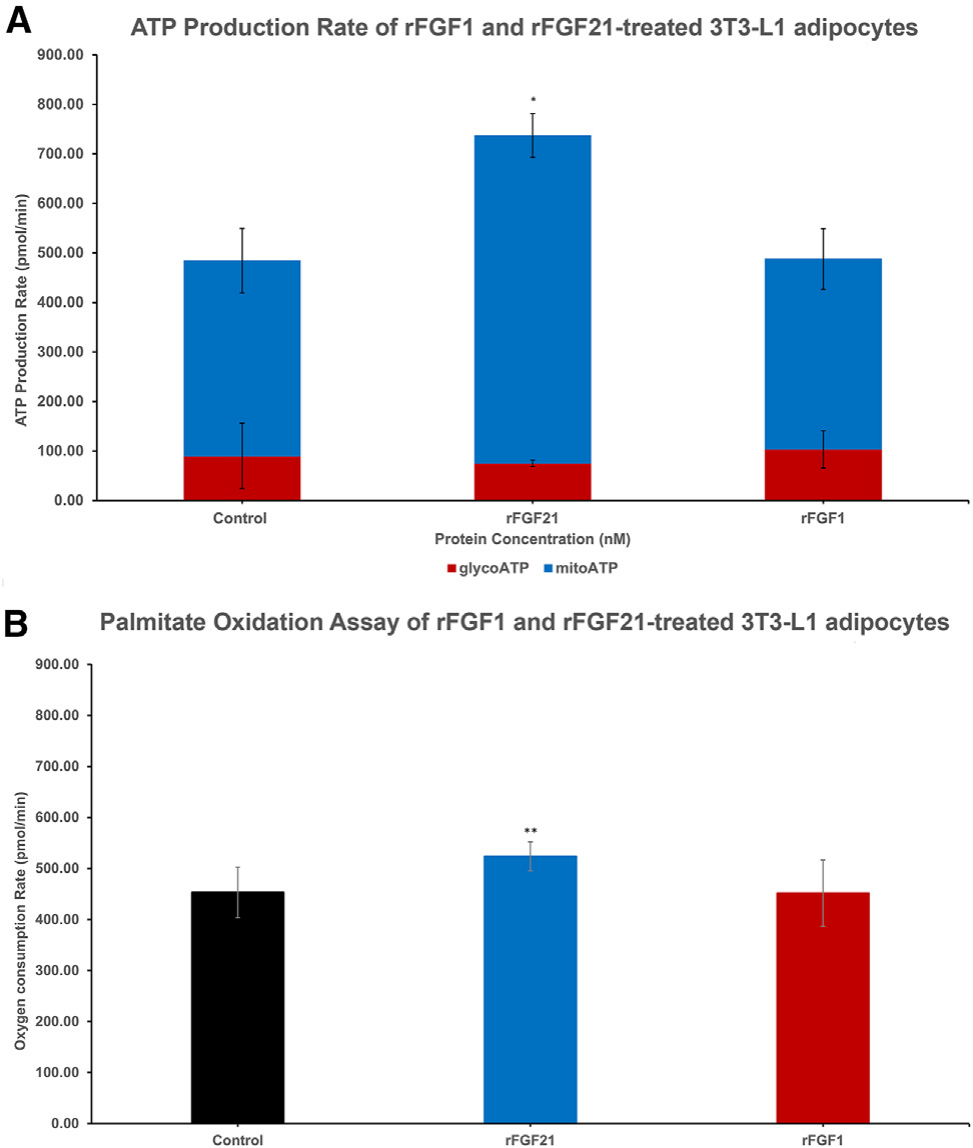
Metabolic activities of rFGF21 and rFGF1 on 3T3-L1 cells. The figure shows the ATP production rate of protein-treated 3T3-L1 adipocytes (at 3.125 nM) in high-glucose media (*A*). On average, rFGF21 showed a higher ATP production rate than control but not rFGF1. The figure also shows the palmitate oxidation activities of both proteins on 3T3-L1 (*B*). rFGF21-treated cells showed a statistically higher palmitate oxidation rate than control, but rFGF1 does not. rFGF21 is in blue and rFGF1 is in red. ∗*p* ≤ 0.05, ∗∗*p* ≤ 0.01, and α∗-value = 0.025.

FGF21 is a well-established metabolic regulator that facilitates glucose uptake in fat cells via glucose transporter-1 upregulation (32). To verify these metabolic effects of rFGF21 and rFGF1, we examine their effects on 3T3-L1 adipocytes in high-glucose media using the Seahorse XF ATP production assay. At a concentration of 3.125 nM, rFGF21-treated adipocytes show enhanced mitochondrial oxidative phosphorylation on average compared to the control group (661.6 ± 44.3 and 394.3 ± 65.1 pmol/min, respectively) (Fig. 10). rFGF21-treated adipocytes also produce more total ATP than the control group on average (736.7 ± 50.4 and 484.2 ± 126.9 pmol/min, respectively) (Fig. 10). These observations suggest that rFGF21 can uptake glucose from high-glucose media to drive the mitochondrial oxidative phosphorylation and increase the total ATP output of adipocytes. This implies that rFGF21 may retain the glucose uptake ability of native FGF21. In contrast, rFGF1 does not demonstrate these effects. However, there are limitations to these experiments. The observed differences in total ATP production between rFGF21 and the control are not statistically significant after adjusting for the α-level (α∗) using the Bonferroni post hoc test, although the *p* value of the *t*-test between rFGF21 and control is less than 0.05. This discrepancy may be due to the low rFGF21 concentration used (3.125 nM) or the small sample size (*n* = 5). Thus, future studies with a larger sample size and testing higher protein concentrations are needed.

Previous studies have shown that FGF21 increases the lipid oxidation rate in the liver (15,16,17). Here, we assess the ability of rFGF21 and rFGF1 to enhance the adipocyte palmitate oxidation rate using the Seahorse XF palmitate oxidation assay. Our findings show that rFGF21-treated adipocytes have a statistically higher lipid oxidation rate (523.6 ± 28.4 pmol/min) than the control group (453.1 ± 49.5 pmol/min) (Fig. 10), suggesting that rFGF21 also promotes exogenous lipid (palmitate) oxidation in adipocytes, similar to its effects in the liver. rFGF1 does not display any differences in its lipid oxidation rate compared to the control.

In summary, our results demonstrate that rFGF21 exhibits significant biological activities in both fibroblasts and adipocytes, especially through mitochondrial functions. Specifically, rFGF21 promotes cell proliferation in NIH/3T3 fibroblasts in a dose-dependent manner and enhances metabolic activities in 3T3-L1 adipocytes, including increased mitochondrial oxidative phosphorylation through glucose uptake from glucose-rich media, an enhanced total ATP production rate, and an increased exogenous lipid (palmitate) oxidation rate. These activities are likely mediated by rFGF21-FGFR1c interaction (43). Since both FGFR1c and KLB are expressed in NIH/3T3 fibroblasts (47,48) and 3T3 L1 differentiated adipocytes (32,42,49,50), rFGF21 may assert its activities in both cell lines, albeit with different outcomes, highlighting the importance of this receptor in FGF21’s biological functions.

### Conclusions

FGF21 has emerged as a promising therapeutic for treating metabolic diseases like metabolic dysfunction-associated steatohepatitis, type 2 diabetes mellitus, and obesity. Although extensive research has been conducted on FGF21’s physiological functions and relevance in metabolic disorders, challenges remain in the production of rFGF21, and biophysical studies on its structure are still incomplete. Herein, we report the production of rFGF21 using the *Rosetta-gami* bacterial expression system, which produces a high yield of soluble and pure products. We also characterize the biophysical properties and functions of rFGF21. The results show that rFGF21 is thermally unstable, prone to trypsin digestion, and has low HS affinity. Circular dichroism spectrophotometry reveals that rFGF21 contains β sheet secondary structures, while intrinsic fluorescence spectrophotometry indicates tyrosine emissions without tryptophan quenching. rFGF21 is easily induced to unfold in urea and possesses a high number of solvent-exposed hydrophobic pockets, as assessed by ANS binding. Furthermore, we report the biological activities of rFGF21, including its mitogenic effects on NIH/3T3 cells and its ability to influence mitochondrial functions by enhancing mitochondrial oxidative phosphorylation and lipid oxidation in 3T3-L1 adipocytes, implicating its role in treating metabolic diseases like diabetes and fatty liver diseases.

In conclusion, our study produces biologically active rFGF21 with high purity and solubility using a bacterial expression system and presents an efficient and cost-effective method to address the challenges associated with FGF21 production. The biophysical and functional characterizations provided here offer valuable insights into FGF21’s properties and its structure-function relationship, as well as an inference of FGF21’s roles in treating metabolic diseases by influencing the mitochondrial functions of fat cells. Furthermore, these findings lay a foundational framework for engineering novel structure-based FGF21 variants with enhanced biological activities and stability to treat metabolic diseases.

## Data and code availability

Data will be made available upon request.

## Acknowledgments

This work is financially supported by the National Institute of General Medical Sciences of the 10.13039/100000002National Institutes of Health (R15GM154267), the National Institute of General Medical Sciences of the National Institutes of Health and the Arkansas Integrative Metabolic Research Center at the 10.13039/100007756University of Arkansas (P20GM139768), the National Institute of General Medical Sciences of the National Institutes of Health and the University of Arkansas Statewide Mass Spectrometry Center (P30GM103450), the 10.13039/100000015Department of Energy (DE-FG02-01ER15161), and the University of Arkansas Honors College. T.K.S.K. is the Mildred-Cooper Chair of Bioinformatics and would like to gratefully acknowledge this endowment grant.

## Author contributions

Writing – original draft, P.P. and J.H.; writing – review & editing, P.P., J.H., and T.K.S.K.; investigation, P.P. and J.H.; data curation, P.P. and J.H.; formal analysis, P.P.; methodology, P.P. and T.K.S.K.; validation, P.P.; visualization, P.P. and J.H.; conceptualization, T.K.S.K.; funding acquisition, T.K.S.K.; project administration, T.K.S.K.; supervision, T.K.S.K.; resources, T.K.S.K.

## Declaration of interests

The authors declare no competing interests.

## Supporting material

Document S1. Figures S1–S5

Document S2. Article plus supporting material
