# Supplementary material for: Overexpression and biophysical and functional characterization of a recombinant FGF21
Source: Biophys Rep (N Y). 2025 Jan 29;5(1):100198. doi: 10.1016/j.bpr.2025.100198 (PMC11869967; doi:10.1016/j.bpr.2025.100198)
Supplement: Document S1. Figures S1–S5 [file mmc1.pdf]

Biophysical Reports, Volume *5*

Supplemental information

Overexpression and biophysical and functional characterization of a recombinant FGF21

Phuc Phan, Jason Hoang, and Thallapuranam Krishnaswamy Suresh Kumar

SUPPLEMENTAL MATERIALS AND METHODS

TABLE OF CONTENTS

Figure S1. Materials and compositions of buffers and media.

Figure S2. Transformation of recombinant FGF1 and FGF21 in different *Escherichia coli* hosts.

Figure S3. Expression of recombinant FGF1 and FGF21 *Escherichia coli* hosts.

Figure S4. Solubility of recombinant FGF21 using BL21(DE3) and Rosetta-gami hosts.

Figure S5. Intrinsic fluorescence of folded and unfolded recombinant FGF1 and FGF21.

Figure S1. Materials and compositions of buffers and media.

The bacterial cell lines used in this project are *XL10 gold* (Agilent Technologies, USA), *Rosetta-gami* (Sigma and MP Biomedicals, LLC.), and *BL21 Star* (DE3) (Thermo Fisher Scientific, USA). Luria-Bertani (LB) broth was from IBI Scientific, USA. Chromatography resins (Nickel Sepharose and Heparin Sepharose) were obtained from GE Healthcare, USA. Ethanol, methanol, acetic acid, buffer components (Na_2HPO_4, NaH_2PO_4, NaCl, tris, glycine), acrylamide, bis-acrylamide, Coomassie blue, urea crystal, Tween 20, imidazole (IMD), isopropyl β-D-thiogalactoside (IPTG) and glycerol were supplied by VWR, USA. Sigma and MP Biomedicals, LLC provided phenylmethylsulphonyl fluoride (PMSF), trypsin (from bovine pancreas), 3-isobutyl-1-methylxanthine (IBMX), insulin (bovine), and low molecular weight (~3000 Da) heparin sodium salt. EMD (USA) supplied ammonium sulfate. Abcam, UK supplied the alkaline phosphatase anti-His antibody [HIS-1]. Nitrocellulose membrane 0.2 μm was from Bio-Rad Laboratories, Inc., USA. Mammalian cell lines used here were NIH/3T3 and 3T3-L1 cells (ATCC, USA) and other related reagents (Dulbecco's Modified Essential Medium (DMEM), fetal bovine serum (FBS), bovine calf serum (BCS)) were obtained from ATCC, USA. Penicillin–streptomycin, trypsin in ethylenediaminetetraacetic acid (EDTA) (0.25%), and Hoechst 33342 were from Thermo Fisher Scientific, USA. Dexamethasone was from G Biosciences, USA. Other materials and chemicals were of high-quality analytical grade, as described elsewhere [44]. Samples were in 10 mM phosphate buffer, 150 mM NaCl, and 25 mM $(NH_4)_2SO_4$ at pH 7.2 (working buffer) unless stated otherwise.

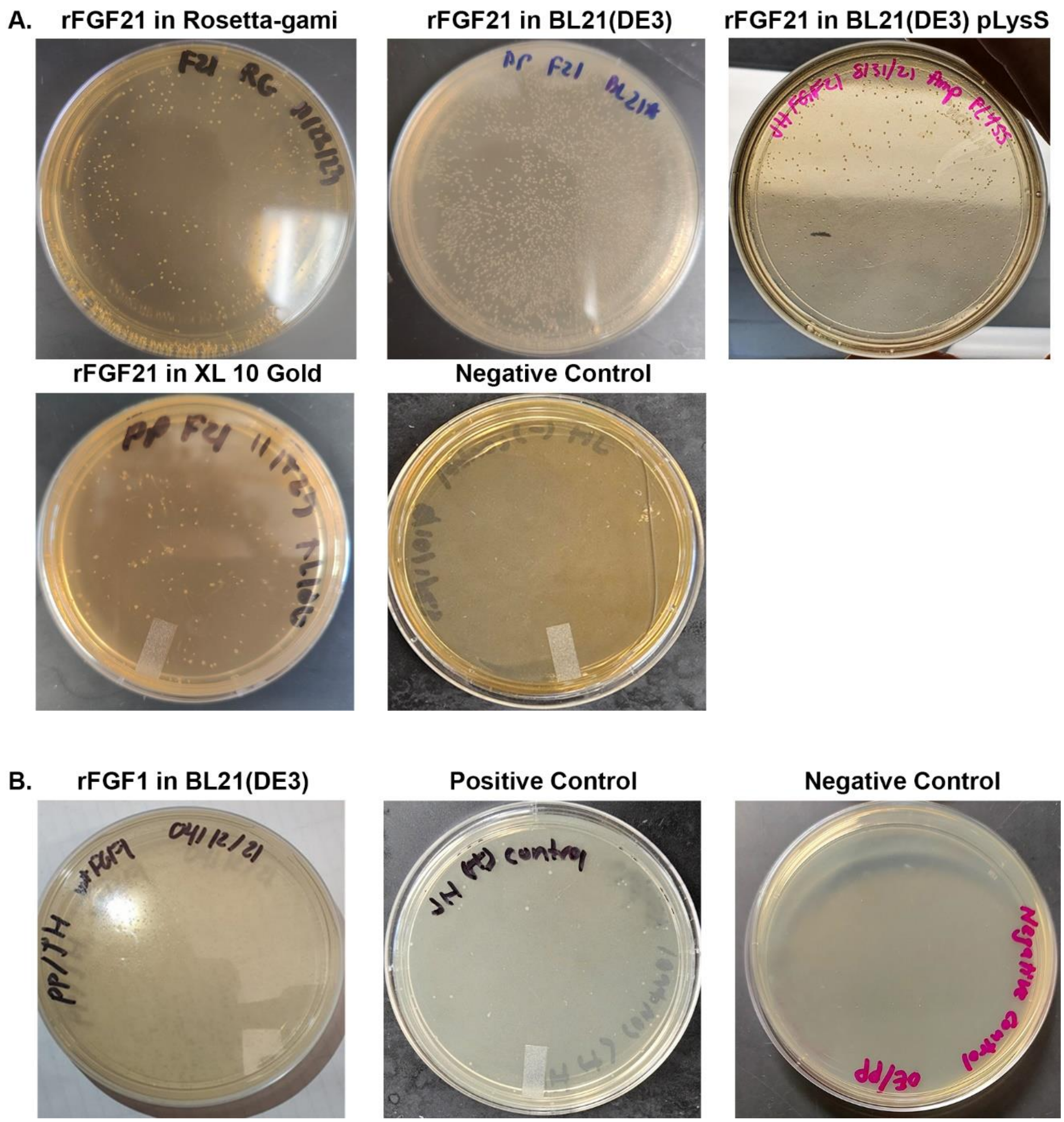

Figure S2. Transformation of recombinant FGF1 and FGF21 in different *Escherichia coli* hosts. Panel A: Different transformed *E. coli* hosts containing recombinant FGF21, including *Rosetta-gami*, *BL21(DE3)*, *XL 10 Gold*, and negative control plate. Panel B: Transformed *BL21(DE3)* bacterial host containing recombinant FGF1.

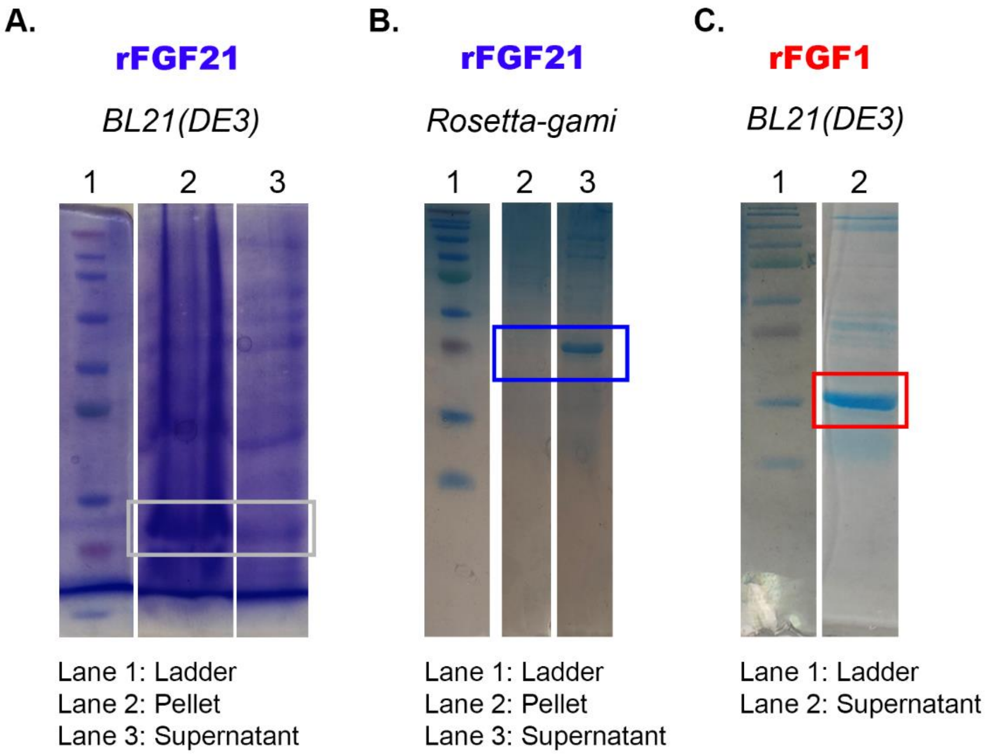

Figure S3. Expression of recombinant FGF1 and FGF21 *Escherichia coli* hosts. Panel A shows IPTG-induced rFGF21 expression in *BL21(DE3)*, which is present mostly in lysate pellet as inclusion body and in small amount in lysate supernatant as soluble protein. Panel B shows IPTG-induced expression of rFGF21 expression in Rosetta-gami, with the majority of product is present in lysate supernatant as soluble protein and in small amount in lysate pellet as inclusion body. Panel C shows IPTG-induced rFGF1 expression in BL21(DE3) as soluble protein in lysate supernatant.

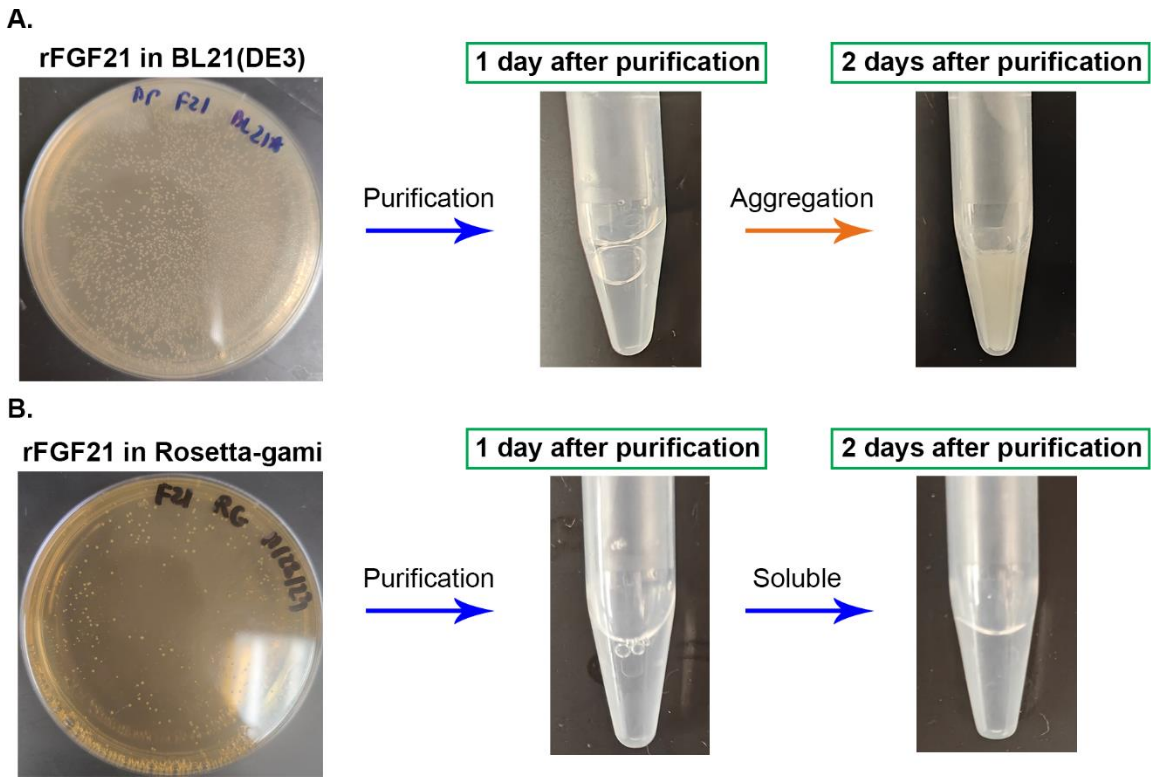

Figure S4. Solubility of recombinant FGF21 using BL21(DE3) and Rosetta-gami hosts. Panel A shows recombinant FGF21-containing BL21(DE3) colonies. The protein produced from these colonies was purified and rapidly aggregated after purification. Panel B shows recombinant FGF21-containing Rosetta-gami colonies. The protein produced from these colonies was purified and remained soluble after purification.

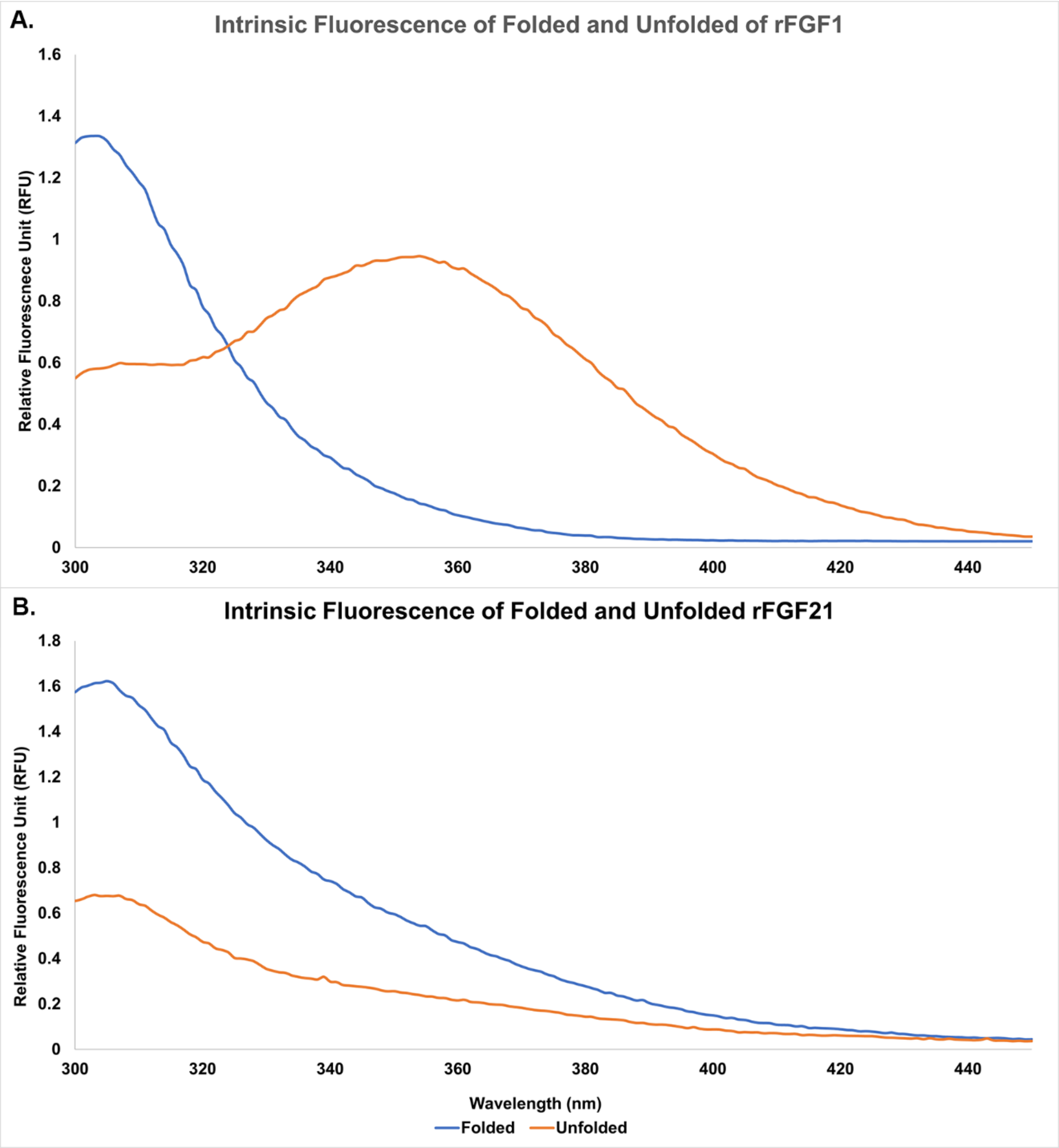

Figure S5. Intrinsic fluorescence of folded and unfolded recombinant FGF1 and FGF21. Panel A shows the intrinsic fluorescence of folded and unfolded rFGF1. Folded rFGF1 shows tyrosine emission at around 308 nm and unfolded rFGF1 shows tryptophan emission at around 350 nm. Panel B shows the intrinsic fluorescence of folded and unfolded rFGF21. Folded and unfolded rFGF21 show tyrosine emission at around 308 nm.
